# Prognostic value of elevated cardiac troponin in aneurysmal subarachnoid hemorrhage: a systematic review and meta-analysis

**DOI:** 10.3389/fneur.2025.1506819

**Published:** 2025-03-18

**Authors:** Jiahui Zhao, Shujuan Gu, Xudong Zhao, Shisong Wang, Qichen Pan, Cunyi Zou

**Affiliations:** Department of Neurosurgery, The First Affiliated Hospital of China Medical University, Shenyang, China

**Keywords:** subarachnoid hemorrhage, cardiac troponin, complication, prognosis, meta-analysis, systematic review

## Abstract

**Objective:**

Subarachnoid hemorrhage (SAH) is a common intracranial hemorrhagic condition associated with a high mortality rate and significant disability due to serious complications. In clinical practice, we have observed that elevated serum cardiac troponin (cTn) levels correlate with a poor prognosis in SAH. Although some studies have reported this correlation, these studies had small sample sizes and did not make a distinction between traumatic SAH and aneurysmal SAH. Accordingly, we aimed to systematically analyze the prognostic evaluation value of elevated cTn levels in SAH by reviewing all existing studies to provide a clinical reference.

**Methods:**

We selected studies on cTn and SAH from PubMed, Medline, Embase, Web of Science, Cochrane Library, and Clinical Trails databases published before December 2023. The Newcastle–Ottawa Scale was used to evaluate the quality of the included studies. PRISMA and AMSTAR guidelines were followed to assess the methodological quality of the systematic review. We divided the included studies into two groups: aneurysmal subarachnoid hemorrhage (aSAH) group and total subarachnoid hemorrhage (toSAH) group. The total subarachnoid hemorrhage (toSAH) group included aSAH and traumatic SAH studies for analysis. The pooled effect size was calculated using R studio and Stata 14.0.

**Results:**

In the toSAH group, 1,559 out of 6,349 (24.55%) SAH patients from 33 studies exhibited elevated cTn levels, while 25.0% in the aSAH group also exhibited similar results. In the aSAH group, high levels of cTn were significantly related to increased mortality both in the hospital [OR = 2.51, 95%CI (1.95, 3.23)] and 3 months later [OR = 2.27, 95%CI (1.47, 3.49)]. An increased incidence of disturbance of consciousness [OR = 2.28, 95%CI (1.41, 3.67)], delayed cerebral ischemia (DCI) [OR = 1.99, 95%CI (1.40, 2.83)], physical disability [OR = 2.39, 95%CI (1.79, 3.20)], cardiac dysfunction [OR = 3.97, 95%CI (2.95, 5.33)], arrhythmias [OR = 4.87, 95%CI (2.52, 9.41)], abnormal ventricular wall motion [OR = 8.20, 95%CI (3.70, 18.18)], and neurogenic pulmonary edema [OR = 2.76, 95%CI (1.85, 4.12)] were associated with elevated cTn levels. In the total SAH patient group, the results were further validated.

**Conclusion:**

Elevated cTn levels were associated with a poor prognosis and an increased risk of adverse events, particularly in aneurysmal SAH. Clinicians should prioritize monitoring SAH patients with elevated cTn levels and consider early intervention strategies.

**Systematic review registration:**

https://www.crd.york.ac.uk/PROSPERO/view/CRD42023433744, identifier: CRD42023433744.

## Introduction

Subarachnoid hemorrhage (SAH) is a common cerebrovascular condition resulting from the rupture of intracranial aneurysms or traumatic brain injury, accounting for 6–10% of stroke cases (1, 2). As a type of cerebral hemorrhage, SAH has garnered significant attention due to its high mortality rate (25–30%) and a considerable incidence of complications (15–20%), such as disturbance of consciousness, physical disability, arrhythmias, and neurogenic pulmonary edema (3, 4). However, there is a lack of effective prognostic indicators for evaluating adverse events in SAH.

Cardiac troponin (cTn) is a specific biomarker used to identify myocardial injury and is commonly employed in the clinical diagnosis of myocardial infarction. Persistently elevated levels of troponin often indicate severe myocardial ischemia. In the diagnosis and treatment of patients with SAH, we observed that many patients presented with elevated troponin levels, which were associated with a poor prognosis and severe complications, such as cerebral vasospasm, myocardial ischemia, and even death. Sahar reported that 33–68% of patients had elevated cTn levels within 48 h after SAH (5). Nevertheless, we found that the majority of SAH patients who had high cTn levels did not actually have myocardial injury. This interesting phenomenon prompted us to explore the mechanism behind elevated cTn levels and the crucial role of serum cTn in SAH.

Growing evidence indicates that subarachnoid hemorrhage causes excessive excitation of the sympathetic nervous system and leads to the release of a large number of catechol hormones, resulting in the contraction of cardiovascular and cerebrovascular vessels (6, 7). As a result, elevated serum cTn levels and a series of SAH complications emerge. In 2015, Zhang first reported that cTn elevation was associated with mortality, disability, and delayed cerebral ischemia (DCI) after SAH through meta-analysis (8). Sahar further supplemented the analysis of cTn and adverse cardiovascular outcomes (5). However, previous studies had small sample sizes and lacked comprehensiveness in studying the complications. Moreover, these studies did not make a distinction between traumatic SAH and aneurysmal SAH (aSAH). Here, we conducted a meta-analysis to explore the predictive value of elevated cardiac troponin for a poor prognosis in aneurysmal subarachnoid hemorrhage (aSAH) and total subarachnoid hemorrhage (toSAH), with the aim of providing a valuable reference for clinical management.

## Methods

The systematic review and meta-analysis adhered to the PRISMA and AMSTAR guidelines (9, 10).

### Search strategy

The literature was searched in Embase, Medline, PubMed, Web of Science, Clinical Trials, and Cochrane Library databases. We used a combination of MeSH terms and free terms to search for literature on the relationship between troponin elevation and complications after subarachnoid hemorrhage. The search query was as follows: “hemorrhage, subarachnoid” OR “aneurysmal subarachnoid hemorrhage” OR “spontaneous subarachnoid hemorrhage” AND “troponin” OR “troponin complex” OR “troponin-I” OR “troponin-T.” The search was completed in January 2024.

### Inclusion criteria

1. Study type: All included literature consisted of retrospective or observational studies, including cohort studies and case–control studies.
2. Study population: Studies involving patients diagnosed with subarachnoid hemorrhage resulting from various etiologies were included. The literature must contain comprehensive patient data, including troponin values, complications, and clinical outcomes.
3. Publication date of the searched articles: To ensure the inclusion of recent literature and to minimize study bias, only articles published from 2000 to 2023 were screened.

### Exclusion criteria

1. Study type: Meta-analyses, systematic reviews, and case reports were excluded.
2. Study population: Studies involving patients with conditions other than subarachnoid hemorrhage and those lacking comprehensive patient information and troponin values were excluded.
3. Study quality: Studies with a Newcastle–Ottawa scale score of less than 5, or those with incomplete data and a small sample size (*N* < 20), were excluded.

### Study indicators and data extraction

We extracted the following data from the included literature: title, year of publication, first author’s name, country and region, study type, number of included patients, troponin levels, and the number of patients experiencing various clinical outcomes (including recovery, death, disturbance of consciousness, delayed cerebral ischemia, neurological impairment, cardiac dysfunction, arrhythmias, abnormal ventricular wall movement, and pulmonary edema). Disability at discharge was defined based on the original literature, either indicating the loss of self-care ability in patients or using the modified Rankin Scale (with scores of 4 and 5) (11). Evaluation of the level of consciousness at discharge was determined using the Glasgow Coma Scale (GCS) or the World Federation of Neurological Surgeons (WFNS) grading system (GCS score < 3 or WFNS > III) (12). In addition, cerebral vasospasm was considered a form of delayed cerebral ischemia. Relevant data on these complications were extracted from the included literature.

### Literature quality evaluation

The Newcastle–Ottawa Scale was employed to assess case–control and cohort studies, as seen in a previous study (13), considering factors such as participant selection, comparability between groups, and outcome assessment (14). The maximum attainable score was 9 points. A score exceeding 5 points indicated good quality. Studies with 5 points or more were included, while those with poor quality were excluded (14).

### Statistical analysis

R studio and Stata 14.0 were used for data analysis. A cross-table was constructed according to the number of patients in each group and the number of patients in the elevated-troponin and unchanged groups to calculate the odds ratio (OR). If the original study had already calculated odds ratios, we utilized the method of combining odds ratios. The Q test was employed to assess heterogeneity. According to standard statistical guidelines, if I^2^ is less than 50% and the *p*-value is greater than 0.05, it indicates no heterogeneity and good consistency. In such cases, we select the fixed-effect model to calculate the odds ratio using the Cochran–Mantel–Haenszel method. However, if there is heterogeneity (I > 50% and *p* < 0.05), we opt for the random-effects model to calculate the odds ratio using the DerSimonian and Laird method. Our study included literature from various countries with differences in patient selection, inclusion criteria, and exclusion criteria, leading to obvious heterogeneity. Therefore, we adopted the random-effects model. To elucidate the different prognostic values of cTn in aSAH and toSAH, we performed subgroup analyses, first in patients with aSAH and then in the toSAH group, including all patients. Subgroup analyses based on study type were conducted to identify the source of heterogeneity. In addition, funnel plots generated using Stata software and Egger’s test were employed to assess publication bias (9). If the funnel plot or Egger’s test indicates publication bias, trim-and fill-analysis should be performed to further evaluate the reliability of the study results.

## Results

### Literature selection and baseline characteristics

**Figure 1:**
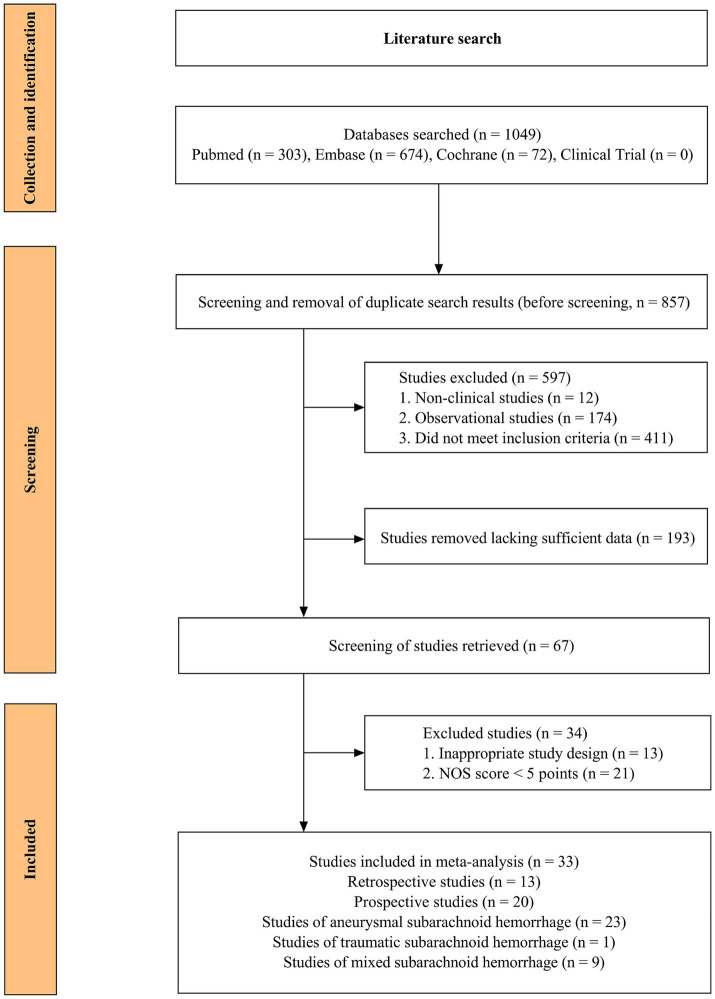
Flow chart (literature search and screening).

After screening 1,049 articles from the initial search, 33 articles (20 prospective studies and 13 retrospective studies) were included in the study (Figure 1) (15–47). The basic information of the included literature, such as author, year, country, sample size, number of patients with elevated levels of cTn, number of patients with various complications, and clinical outcomes, is shown in Table 1. The NOS score of the included literature was greater than 5 (Supplementary Table S1). A total of 6,349 patients with SAH were finally included in this meta-analysis (Supplementary Table S2). Of these, 1,559 (24.55%) patients experienced increased cTn levels across mixed types of SAH. Of 4,156 patients in the aSAH study across 23 articles, 1,038 (25.0%) had elevated cTn levels. This indicated that enhanced cTn levels are a common phenomenon in patients with SAH, warranting further research, especially in aSAH due to its pathogenesis.

**Table 1:** Basic information of the included studies.

Author	Country	Type	Objective	Total	Follow-up duration	Troponin elevated	Cardiac dysfunction	DCI	Pulmonary edema	Level of consciousness	Physical disability	Death
Lin et al. (15)	China	R	A	213	34.3 months	55/213	30	39	NR	NR	60	20
Anetsberger et al. (16)	Germany	P	A	106	3 months	36/106	10	22	24	NR	38	14
Bender et al. (17)	Germany	R	T	117	In hospital	59/117	NR	NR	34	NR	NR	NR
Alkhachroum et al. (18)	USA	R	M	169	In hospital	48/169	NR	NR	NR	NR	53	21
Guette et al. (19)	France	P	M	137	3 months	76/137	NR	NR	NR	NR	30	43
Akkermans et al.(20)	The Netherlands	P	A	159	12 months	43/159	11	NR	NR	40	NR	11
Nastasovic et al. (21)	Serbia	P	A	262	In hospital	82/262	NR	NR	19	NR	NR	NR
Mahmoud et al. (22)	USA	R	A	244	In hospital	50/244	NR	NR	NR	NR	NR	38
Duello et al. (23)	USA	R	A	175	1 month	36/175	NR	NR	NR	NR	NR	32
Bilt et al. (24)	The Netherlands	P	A	301	In hospital	97/301	58	NR	NR	NR	NR	NR
Bilt et al. (25)	The Netherlands	P	A	300	3 months	96/261	NR	117	NR	NR	55	58
Ahmadian et al. (26)	USA	R	A	617	In hospital	48/67	NR	16	NR	NR	31	20
Gupte et al. (27)	USA	R	A	225	In hospital	47/201	NR	48	NR	NR	30	51
Degos et al. (28)	USA	P	A	368	12 months	64/364	NR	NR	NR	NR	NR	NR
Matthew et al. (29)	USA	R	M	110	6 months	38/100	NR	NR	NR	NR	26	20
Miketic et al. (30)	USA	P	A	239	3 months	80/239	NR	NR	NR	NR	53	NR
Ichinomiya et al. (31)	Japan	P	A	71	In hospital	8/71	NR	39	NR	NR	41	15
Chung et al. (32)	Korea	R	M	253	1 month	28/253	NR	NR	NR	55	NR	25
Hravnak et al. (33)	USA	P	A	204	3 months	64/204	19	NR	NR	45	36	NR
Jeon et al. (34)	Korea	R	A	114	In hospital	34/114	NR	32	NR	NR	52	NR
Thomas et al. (35)	USA	P	A	171	3 months	33/171	NR	NR	9	NR	NR	NR
Sandhu et al. (36)	USA	P	M	96	In hospital	20/96	NR	NR	NR	NR	NR	16
Tanabe et al. (37)	USA	P	A	103	In hospital	54/103	17	NR	33	NR	NR	NR
Ramappa et al. (38)	USA	R	A	83	In hospital	31/83	9	NR	NR	NR	19	31
Pereira et al. (39)	France	P	A	51	12 months	24/51	NR	NR	NR	NR	22	NR
Hays et al. (40)	USA	R	M	235	In hospital	38/235	NR	NR	NR	NR	NR	90
Kothavale et al. (41)	USA	P	M	300	In hospital	NR	NR	NR	NR	NR	NR	NR
Sirisha et al. (42)	USA	P	M	300	In hospital	52/300	NR	NR	NR	NR	NR	38
Naidech et al. (43)	USA	R	M	253	3 months	172/253	NR	45	NR	NR	NR	NR
Schuiling et al. (44)	The Netherlands	P	A	68	3 months	36/68	39	NR	19	NR	40	NR
Kopelnik et al. (45)	USA	P	A	223	In hospital	NR	146	NR	44	NR	NR	NR
Deibert et al. (46)	USA	P	A	43	In hospital	12/43	7	19	NR	12	NR	8
Parekh et al. (47)	Australia	P	A	39	7 days	8/39	5	16	NR	14	NR	NR

Prospective study: R, Retrospective study; A, Aneurysmal subarachnoid hemorrhage; T, Traumatic subarachnoid hemorrhage; M, Mixed; In-hospital means the patients were not followed up after discharge and that only the course of disease and data collected during hospitalization were recorded; DCI, Delayed cerebral ischemia; Level of consciousness refers to the patient’s state of consciousness upon admission, as assessed by the GCS score or WFNS score. NR, no referring.

### Mortality in aSAH

**Figure 2:**
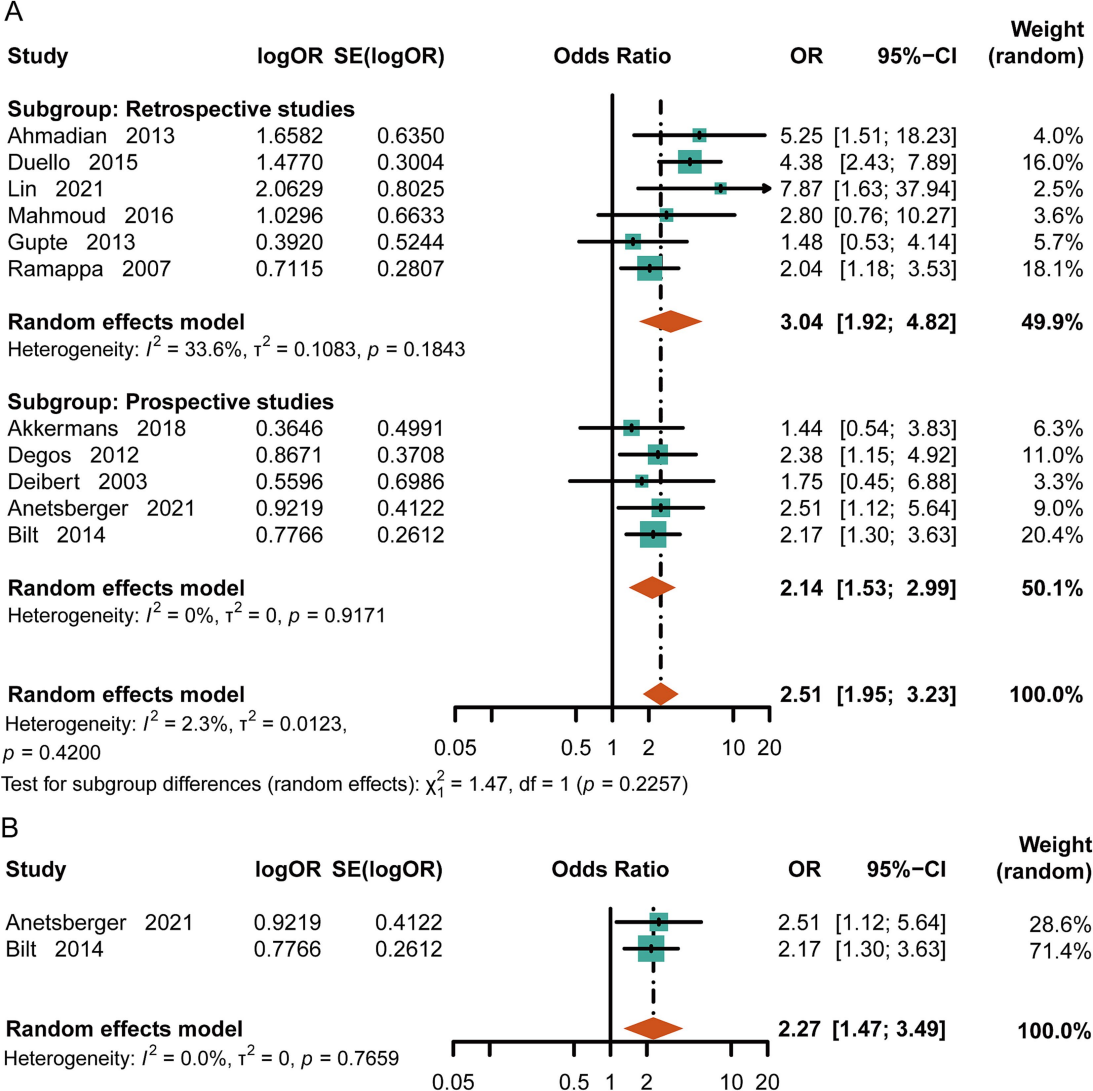
Meta-analysis of mortality in aSAH. **(A)** In-hospital mortality in patients with subarachnoid hemorrhage. **(B)** Mortality at the three-month follow-up.

Comparing the in-hospital mortality rate between the normal-cTn group and the high-cTn group, we found that the mortality rate in the elevated-cTn group was significantly higher [OR = 2.51, 95%CI (1.95, 3.23), *p* < 0.00001, I^2^ = 2.3%] (Figure 2A) in aSAH. Moreover, 3-month mortality after discharge was strongly associated with an elevated level of cTn [OR = 2.27, 95%CI (1.47, 3.49), *p* = 0.0002, I^2^ = 0] (Figure 2B). These results suggest that high levels of cTn may be associated with various adverse complications, leading to a poor prognosis in aSAH.

### Neurologic complications in aSAH

**Figure 3:**
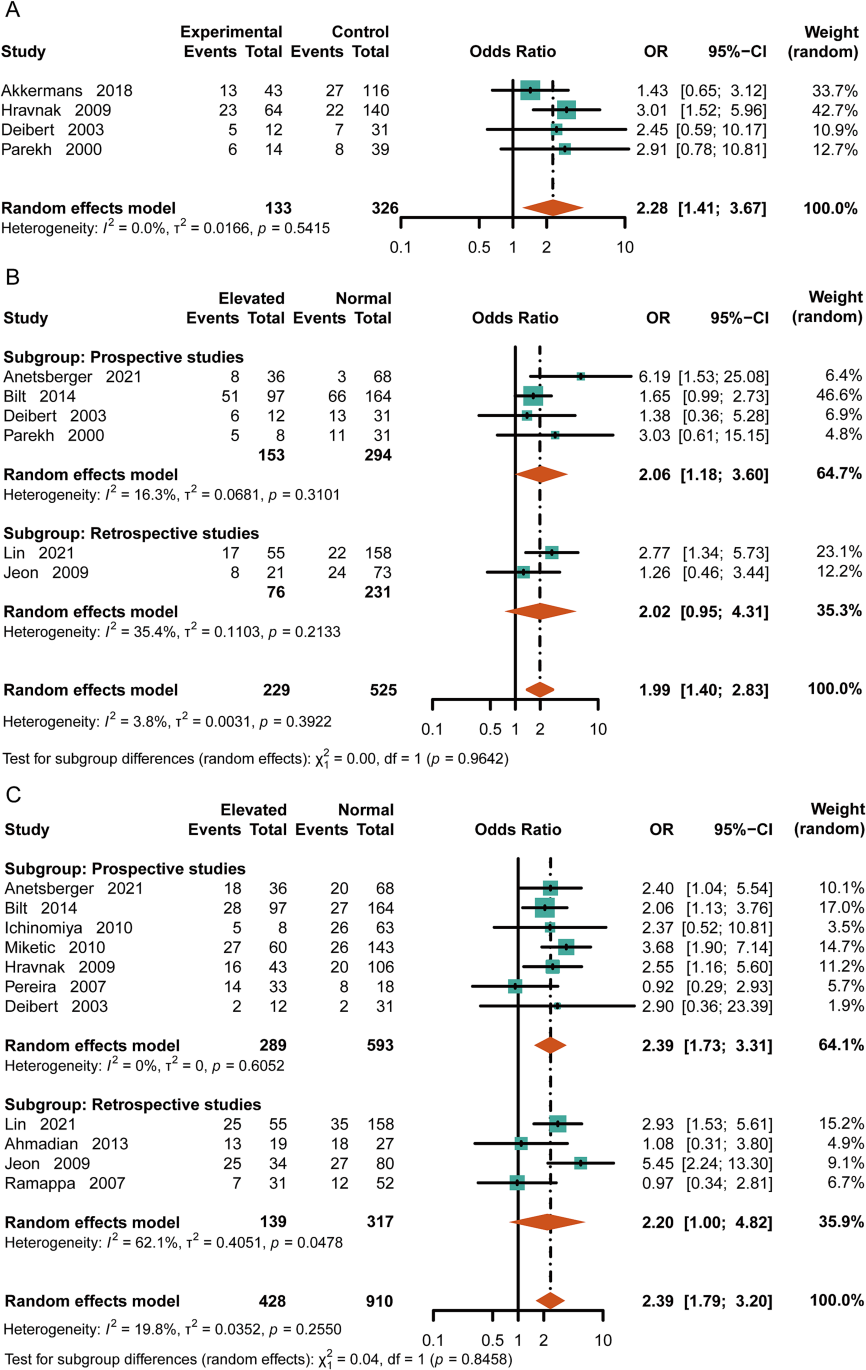
Risk of neurologic complications with high cTn. **(A)** Loss of consciousness at discharge (GCS < 9 or WFNS 4–5). **(B)** Delayed cerebral ischemia or cerebral vasospasm. **(C)** Disability in patients with subarachnoid hemorrhage (modified Rankin Scale score of 4–5).

SAH often leads to serious neurological complications. To evaluate the relationship between cTn levels and consciousness, we extracted data on loss of consciousness (GCS < 9 or WFNS 4–5) from the original literature for patients at discharge. Patients with elevated cTn levels had poorer consciousness upon discharge compared to the control group [OR = 2.28, 95%CI (1.41, 3.67), *p* = 0.0007, I^2^ = 0] (Figure 3A). Meanwhile, the patients with high-cTn levels had an increased risk of developing cerebral vasospasm or delayed cerebral ischemia (DCI) [OR = 1.99, 95%CI (1.40, 2.83), *p* = 0.0001, I^2^ = 3.8%] (Figure 3B). Considering limb movements, we found a positive correlation between elevated cTn levels and physical disability at discharge [OR = 2.39, 95%CI (1.79, 3.20), *p* < 0.0001, I^2^ = 19.8%] (Figure 3C).

### Cardiac dysfunction and neurogenic pulmonary edema in aSAH

**Figure 4:**
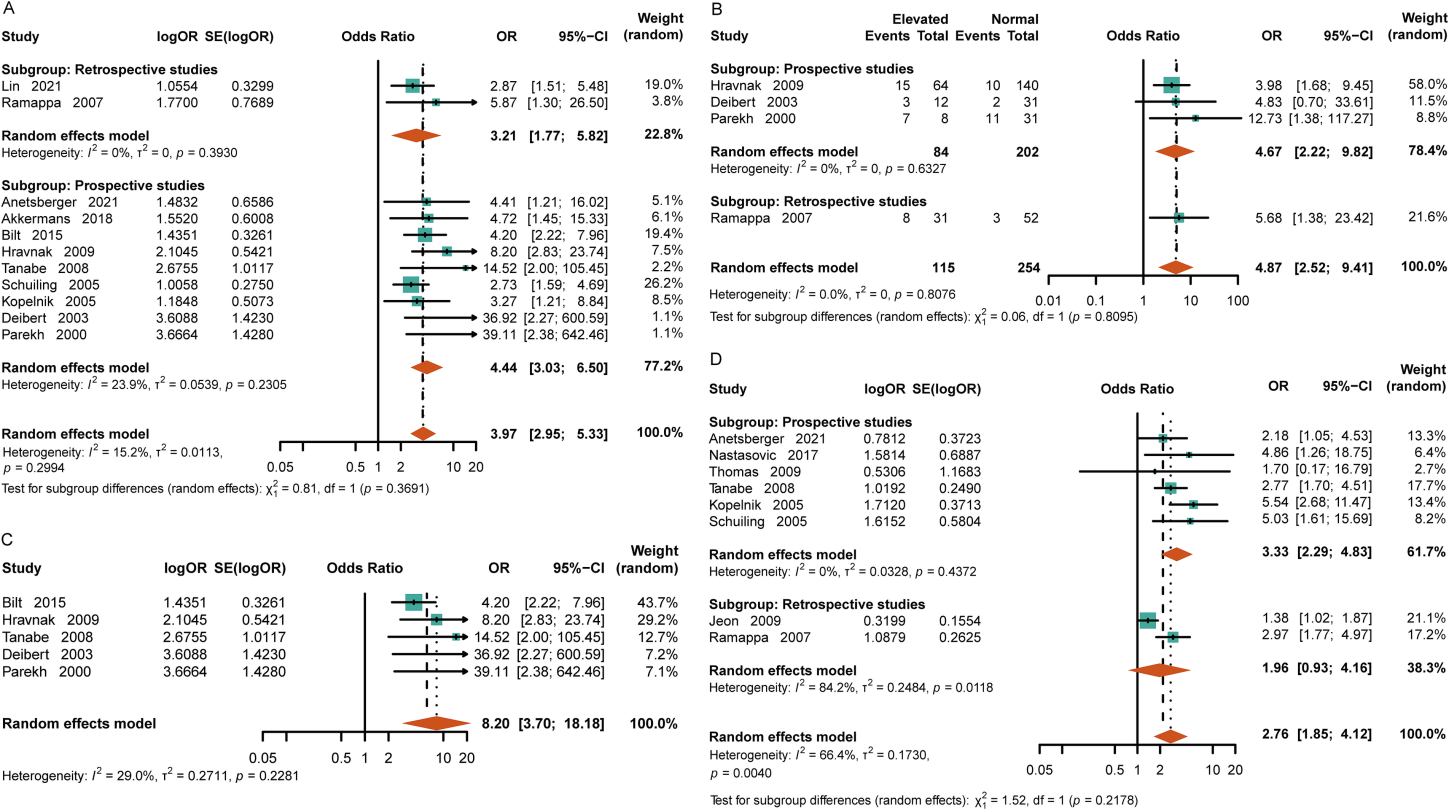
Relationship between cardiopulmonary adverse events and elevated cTn in the patients with aSAH. **(A)** Cardiac dysfunction, including arrhythmia, acute myocardial infarction, heart failure, and echocardiographic evidence of abnormal ventricular wall movement. **(B)** Arrhythmias, including atrial fibrillation, ventricular fibrillation, and premature beats. **(C)** Echocardiographic evidence of abnormal ventricular wall motion after SAH. **(D)** Neurogenic pulmonary edema.

Compared to the normal-cTn group, the patients with elevated cTn levels were more likely to experience cardiac dysfunction after SAH [OR = 3.97, 95%CI (2.95, 5.33), *p* = <0.0001, I^2^ = 15.2%] (Figure 4A). Based on the original literature, we further classified cardiac dysfunction into arrhythmia and ventricular wall motion abnormality groups. Upon analysis, it was found that the patients with increased levels of cTn had a higher incidence of emerging arrhythmia events [OR = 4.87, 95%CI (2.52, 9.41), *p* < 0.0001, I^2^ = 0] (Figure 4B). The likelihood of experiencing abnormal ventricular wall motion in the elevated-cTn group after SAH was 6.59 times higher than that in the control group [OR = 8.20, 95%CI (3.70, 18.18), *p* <0.00001, I^2^ = 29%] (Figure 4C). Furthermore, increased cTn levels were associated with neurogenic pulmonary edema [OR = 2.76, 95%CI (1.85, 4.12), *p* < 0.0001, I^2^ = 66.4%] (Figure 4D).

### Complications in toSAH

To validate the prognostic value of elevated levels of cardiac troponin in patients with toSAH, we conducted an analysis of all 33 articles on SAH. The in-hospital mortality and 3-month mortality after discharge were significantly higher in the elevated-cTn group compared to the control group [OR = 2.60, 95% CI (2.10–3.21), *p* < 0.00001, I^2^ = 13.9%; OR = 2.36, 95%CI (1.61–3.48), *p* < 0.0001, I^2^ = 0] (Supplementary Figures S1A,B). In patients with toSAH, cTn appeared to have a higher association with disturbance of consciousness at admission [OR = 2.09, 95%CI (1.40, 3.11), *p* = 0.0004, I^2^ = 0] (Supplementary Figure S1C). The likelihood of DCI [OR = 2.27, 95%CI (1.55, 3.30), *p* < 0.0001, I^2^ = 16.3%] and post-hemorrhage disability [OR = 1.95, 95%CI (1.42, 2.67), *p* < 0.00001, I^2^ = 40.2%] in the patients with elevated cTn levels was significantly higher compared to those with normal cTn levels (Supplementary Figures S1D,E). Moreover, we analyzed the relationship between elevated cTn levels and cardiac dysfunction after SAH and found that the incidence of cardiac dysfunction was significantly greater in the higher-cTn group [OR = 4.34, 95%CI (3.15, 5.98), *p* = 0.24, I^2^ = 20.3%] (Supplementary Figure S1F). There was a strong positive correlation between increased cTn levels and arrhythmia [OR = 4.87, 95%CI (2.52, 9.41), *p* < 0.00001, I^2^ = 18.9%] and ventricular wall motion abnormality [OR = 8.12, 95%CI (4.21, 15.69), *p* = 0.0001, I^2^ = 0%] (Supplementary Figures S1G,H). The patients with elevated cTn levels were prone to experiencing neurogenic pulmonary edema [OR = 2.55, 95%CI (1.82, 3.56), *p* = 0.0001, I^2^ = 58.2%] (Supplementary Figure S1I).

### Heterogeneity and sensitivity analysis

Due to the varying inclusion and exclusion criteria across the included articles from different regions, and to enhance the reliability of our analysis, we used the random-effects model to account for heterogeneity in patient populations. Each group was divided into a prospective study group and a retrospective study group according to the type of the original literature. All the studies showed no obvious heterogeneity and had good sensitivity (Supplementary Figure S2). However, we found significant heterogeneity in the analysis of the relationship between the patients with elevated troponin levels and neurogenic pulmonary edema (aSAH, I^2^ = 66.4%; toSAH, I^2^ = 58.2%) (Figure 4D; Supplementary Figure S1I). To explore the source of heterogeneity, the prospective and retrospective studies were analyzed separately. We found minimal heterogeneity in the prospective studies and significant heterogeneity in the retrospective studies. Combined with the results of other subgroup analyses, we determined that the source of heterogeneity was mainly from the included retrospective studies.

### Assessment of bias

We assessed the results for bias by performing separate funnel plots for each subgroup and conducting Egger’s test. According to the funnel plot and test results, there was no obvious publication bias in our study (Supplementary Figures S3, S4; Supplementary Table S3).

## Discussion

Subarachnoid hemorrhage (SAH), a type of intracranial hemorrhagic condition, carries significant risks of severe neurological and systemic complications, especially in aneurysmal SAH. As an acute stroke event, aSAH is often associated with high mortality and disability rates. Consciousness disorders, delayed cerebral ischemia, vascular spasms, and cardiopulmonary complications are common adverse events in SAH. However, there is still no effective predictive marker for poor prognosis. In this study, we found that 25.0% of the patients in the aSAH group had elevated levels of cTn. Patients with increased cTn levels had a high risk of experiencing disturbance of consciousness, delayed cerebral ischemia, physical disability, cardiac dysfunction, arrhythmias, abnormal ventricular wall motion, and neurogenic pulmonary edema, as confirmed by the meta-analysis of 33 studies (20 prospective studies and 13 retrospective studies) involving 6,349 patients (15–47). This indicated that there was a pathophysiological connection between elevated levels of cTn and adverse events in SAH.

The mainstream view of the pathophysiological mechanism in SAH is that subarachnoid hemorrhage stimulates the sympathetic nervous system, mainly in the insular cortex (48). It activates the sympathetic nervous pathway to release a large amount of catecholamine hormones, including norepinephrine, epinephrine, and dopamine, which may lead to cardiocerebral vasoconstriction and cardiocerebral ischemic events (48, 49). Cerebral vasospasm can lead to a decreased level of consciousness, physical disability, or even cerebral infarction with further damage. Myocardial ischemia results in elevated serum cTn levels, manifesting as angina or acute myocardial infarction. Vasoconstriction and left ventricular diastolic dysfunction can cause an increase in circulating blood volume, resulting in cardiopulmonary complications (49). In addition, the activation of the hypothalamic–pituitary–adrenal axis in SAH also plays a certain role (46). Thus, it can be seen that high cTn levels and complications after SAH are strongly interconnected, explaining the prognostic value of cTn.

We further confirmed that increased cTn levels were strongly associated with mortality and related complications, not only in SAH with mixed etiology but also in aneurysmal SAH. In traumatic patients, death and disability are mainly related to primary brain injury and secondary intracranial hematoma, rather than subarachnoid hematoma. Therefore, we believe that elucidating the predicted value of cTn in aSAH is highly valuable. The main risk factors for aneurysmal SAH include age over 70 years, the location of the ruptured middle cerebral artery aneurysm, Hunt–Hess level of 4–5, aneurysm diameter greater than 7 mm, early aneurysm re-rupture (within 3 months), intraventricular hemorrhage, acute hydrocephalus, and cerebral vasospasm (50). In addition, you reported that the extent of cTn elevation after SAH was related to Hunt–Hess grading and the size of the ruptured aneurysms (51). These findings suggest that high cTn levels play a significant role in determining the prognosis in aSAH. Elevated cTn levels were also found as an independent risk factor for all-cause mortality in aSAH (19). We verified that high cTn levels were associated with a higher likelihood of mortality, disturbance of consciousness, disability dysfunction, and neurogenic pulmonary edema, which should be recognized by clinicians. In addition, the risk factors for adverse events in aSAH, such as age, Hunt–Hess grading, the level of cTn, acute hydrocephalus, and intraventricular hemorrhage, could be collected and aggregated to accurately assess the prognosis. We believe that the extent of cTn elevation will improve risk stratification in SAH patients, which requires further quantitative analysis based on original clinical data.

Unlike previous studies, we classified cardiac dysfunction into arrhythmia and abnormal ventricular wall movement and found that the main types of arrhythmia after SAH were atrial fibrillation and prolonged QT interval. We also found that elevated cTn can predict arrhythmia and abnormal ventricular wall movement, complementing past research (24). Abnormal ventricular wall motion is one of the most common cardiac complications observed after SAH on echocardiography. Left ventricular diastolic dysfunction can lead to neurogenic pulmonary edema, delayed cerebral ischemia, or cerebral vasospasm. Most notably, Takotsubo syndrome, known as stress cardiopathy, occurs in 2 to 15% of SAH cases and refers to temporary local left ventricular systolic and diastolic dysfunction caused by severe emotional or physical stress (52, 53). Caused by the secretion of adrenal hormones from the adrenal medulla, myocardial ischemia occurs without coronary artery stenosis. Due to its self-limitation and good prognosis, doctors should differentiate it from myocardial infarction caused by coronary atherosclerosis.

Clinical management should begin as soon as possible after subarachnoid hemorrhage. When accompanied by high cTn levels, treatment and monitoring should be more comprehensive. Continuous monitoring of arterial blood pressure, heart rate, oxygen saturation, and central venous pressure is required (7). Frequent ECGs and cardiac and cerebral vascular ultrasounds should be performed to evaluate adverse events. Angiotensin-converting enzyme inhibitors (ACEIs), angiotensin receptor blockers (ARBs), and *β*-blockers are potential treatment options for SAH (54). Previous studies have suggested that β-receptor blockers can improve the prognosis and effectively alleviate cerebral vascular insufficiency and left ventricular dysfunction caused by subarachnoid hemorrhage (55). However, if QT interval extension and atrioventricular block occur, β receptor blockers are not recommended for use. We believe β-receptor blockers could be selectively applied in patients with increased cTn. Moreover, adrenaline, noradrenaline, dobutamine, milrinone, and isoproterenol should be avoided because they may exacerbate myocardial ischemia. For patients with heart failure, diuretics and vasodilators, such as nitroglycerin, should be used. If atrial fibrillation occurs, timely administration of amiodarone or electro-conversion is necessary to prevent thrombus detachment and cerebral infarction. Mannitol should be used to reduce intracranial pressure, and nimodipine should be used to alleviate cerebral vasospasm. The explanation of the condition and poor prognosis to the families of patients with SAH and elevated cTn should be more targeted. Meanwhile, clinicians should intensify monitoring and consider early intervention strategies for high-risk adverse complications.

This meta-analysis incorporated several methodological advancements compared to previous studies, including a comprehensive analysis of more complications reported in recent literature and a comparative analysis of the predictive ability of cardiac troponin (cTn) between aneurysmal subarachnoid hemorrhage (aSAH) and overall SAH cases. Nevertheless, there are still several limitations in this meta-analysis. First, due to the incomplete original data from the various literature sources, we could not perform a hierarchical analysis, such as dividing the data by gender, age, BMI, and underlying diseases. Second, as this study was a qualitative analysis, it was difficult to determine a specific critical value for cTn elevation. We were unable to analyze the quantitative relationship between elevated cTn levels and the incidence of complications. In addition, other complications after SAH require further investigation. Third, due to the limited original literature on traumatic SAH, we were unable to analyze the relationship between increased cTn levels and prognosis in traumatic SAH. However, we believe that for traumatic patients, primary brain injury and secondary hematoma play a more significant role in determining the prognosis than SAH itself. Finally, as the included studies were from different countries with varying inclusion criteria, the results might have been overestimated after the meta-analysis. However, we used subgroup analysis, heterogeneity analysis, and bias testing to present the most reliable results possibly. We look forward to more clinical research in the future to fill the gap in this field, allowing us to gain a more comprehensive understanding of the predictive value of cTn in SAH, especially aneurysmal SAH.

## Conclusion

This meta-analysis indicated a significant positive correlation between elevated cTn levels and adverse outcomes in SAH, including mortality, consciousness disorders, DCI, disability, cardiac dysfunction, and neurogenic pulmonary edema. High levels of cTn play a significant role in predicting the prognostic value in aneurysmal SAH. Cardiac troponin levels should be routinely monitored in patients with SAH, and clinicians should assess the risk of related complications based on elevated cTn levels and provide timely treatment.

## Data Availability

The original contributions presented in the study are included in the article/Supplementary material, further inquiries can be directed to the corresponding authors.
