# Supplementary material for: Prognostic value of elevated cardiac troponin in aneurysmal subarachnoid hemorrhage: a systematic review and meta-analysis
Source: Front Neurol. 2025 Mar 18;16:1506819. doi: 10.3389/fneur.2025.1506819 (PMC11960501; doi:10.3389/fneur.2025.1506819)
Supplement: Supplementary file 1 [file Data_Sheet_1.pdf]

Table S1. The NOS quality evaluation of included studies.

Author	Year	Type	Selection	Comparability	Outcome	Points
Lin	2021	Retrospective	★★★★	★★	★★	8
Anetsberger	2021	Prospective	★★★	★★	★★★	8
Bender	2020	Retrospective	★★★	★	★★	6
Alkhachroum	2019	Retrospective	★★★★	★★	★★★	9
Guette	2019	Prospective	★★★	★★	★★	7
Akkermans	2018	Prospective	★★★	★★	★★★	8
Nastasovic	2017	Prospective	★★★★	★★	★★★	9
Mahmoud	2016	Retrospective	★★★	★	★★	6
Duello	2015	Retrospective	★★★	★★	★	6
Bilt	2015	Prospective	★★★	★	★★	6
Bilt	2014	Prospective	★★★	★★	★★	7
Ahmadian	2013	Retrospective	★★★	★★	★★	7
Gupte	2013	Retrospective	★★★	★★	★	6
Degos	2012	Prospective	★★★	★★	★★	7
Matthew	2010	Retrospective	★★★	★★	★★	7
Miketic	2010	Prospective	★★★★	★★	★★	8
Ichinomiya	2010	Prospective	★★★	★★	★★★	8
Chung	2009	Retrospective	★★★★	★★	★★★	9
Hravnak	2009	Prospective	★★★★	★★	★★	9
Jeon	2009	Retrospective	★★★★	★★	★★	8
Thomas	2009	Prospective	★★★★	★★	★★	8
Sandhu	2008	Prospective	★★★★	★★	★★★	9
Tanabe	2008	Prospective	★★★	★★	★	6
Ramappa	2007	Retrospective	★★★	★★	★★★	8
Pereira	2007	Prospective	★★★	★★	★★	7
Hays	2006	Retrospective	★★★	★★	★★	7
Kothavale	2006	Prospective	★★	★★	★★	6
Sirisha	2006	Prospective	★★★	★★	★	6
Naidech	2005	Retrospective	★★★	★★	★	6
Schuiling	2005	Prospective	★★★★	★★	★★	8
Kopelnik	2005	Prospective	★★★★	★★	★★	8
Deibert	2003	Prospective	★★★★	★★	★★★	9
Parekh	2000	Prospective	★★★	★★	★★★	8

Table S2. Patient characteristic of included studies.

Author	Year	Country	Type	Female (%)	Mean age	Smoker (%)	Hypertension (%)	Diabetes mellitus (%)	Heart disease (%)	Pulmonary disease (%)
Lin	2021	China	R	131(56.7)	57	NA	131(61.5)	17(8.0)	33(15.5)	NA
Anetsberger	2021	Germany	P	81(76.0)	55.4	32(30）	50(47)	5(5)	8(8)	11(10)
Bender	2020	Germany	R	113(39.2)	59.9	NA	115(39.9)	NA	NA	34(11.4)
Alkhachroum	2019	USA	R	81(65.9)	NA	NA	NA	NA	9(7.3)	NA
Guette	2019	France	P	95(69)	54	59(43)	39(28)	NA	2(1)	NA
Akkermans	2018	The Netherlands	P	126(79.2)	NA	74(46.5)	46(28.9)	6(3.8)	19(11.9)	NA
Nastasovic	2017	Serbia	P	162(61.8)	NA	62(23.7)	100(38.2)	46(17.6)	68(26.0)	NA
Mahmoud	2016	USA	R	161(66)	59	N	173(71)	49(20)	41(17)	NA
Duello	2015	USA	R	98(56)	57	64(37)	97(56)	NA	15(8)	NA
Bilt	2015	The Netherlands	P	210(70)	57	115(38)	90(30)	14(5)	19(6)	NA
Ahmadian	2013	USA	R	76(87.4)	65	N	64(73.6)	7(8)	27(31.0)	NA
Gupte	2013	USA	R	151(67.1)	57.3	94(41.8)	115(51.1)	29(12.9)	11(4.9)	NA
Degos	2012	USA	P	134(36.4)	50	NA	NA	NA	NA	NA
Matthew	2010	USA	R	53(48.2)	54.5	NA	31(28.2)	NA	NA	NA
Miketic	2010	USA	P	172(71.9)	NA	NA	NA	NA	NA	NA
Ichinomiya	2010	Japan	P	50(70)	70	NA	37(54)	1(1)	NA	NA
Chung	2009	Korea	R	100(39.5)	63.54	138(54.5)	139(54.9)	65(25.7)	NA	NA
Hravnak	2009	USA	P	143(70)	58	69(33.7)	100(49)	19(9.5)	11(5.5)	31(15)
Jeon	2009	Korea	R	84(73.7)	61.1	NA	43(37.7)	NA	NA	65(57.0)
Sandhu	2008	USA	P	63(66)	51	26(27)	56(58)	8(8)	NA	NA
Ramappa	2007	USA	R	50(60)	58.7	42(50)	58(69)	9(10)	12(14)	36(43)
Pereira	2007	France	P	31(60.8)	54	15(29.4)	18(35.3)	1(2)	1(2)	NA
Hays	2006	USA	R	128(54.5)	66	NA	132(73.1)	NA	NA	NA
Kothavale	2006	USA	P	140(68)	54	90(43)	83(40)	17(8)	28(14)	NA
Sirisha	2006	USA	P	207(69)	55	127(42)	126(42)	NA	NA	NA
Naidech	2005	USA	R	308(69.1)	55	260(58.9)	241(48.5)	NA	NA	NA
Schuiling	2005	The Netherlands	P	53(78)	NA	NA	NA	NA	NA	19(28)
Kopelnik	2005	USA	P	140(68)	54	90(43)	83(40)	17(8)	28(14)	NA
Deibert	2003	USA	P	29(67.5)	53	28(63)	26(57.5)	NA	NA	NA
Parekh	2000	Australia	P	24(61.5)	54	NA	NA	NA	NA	NA

Table S3. The results of the Egger test and trim and fill analysis.

Data	P value	Bias (Yes or No)	Trim and fill analysis
Mortality	0.564	No	-
Level of consciousness	0.913	No	-
DCI	0.479	No	-
Disability	0.206	No	-
Cardiac dysfunction	0.301	No	-
Pulmonary edema	0.958	No	-

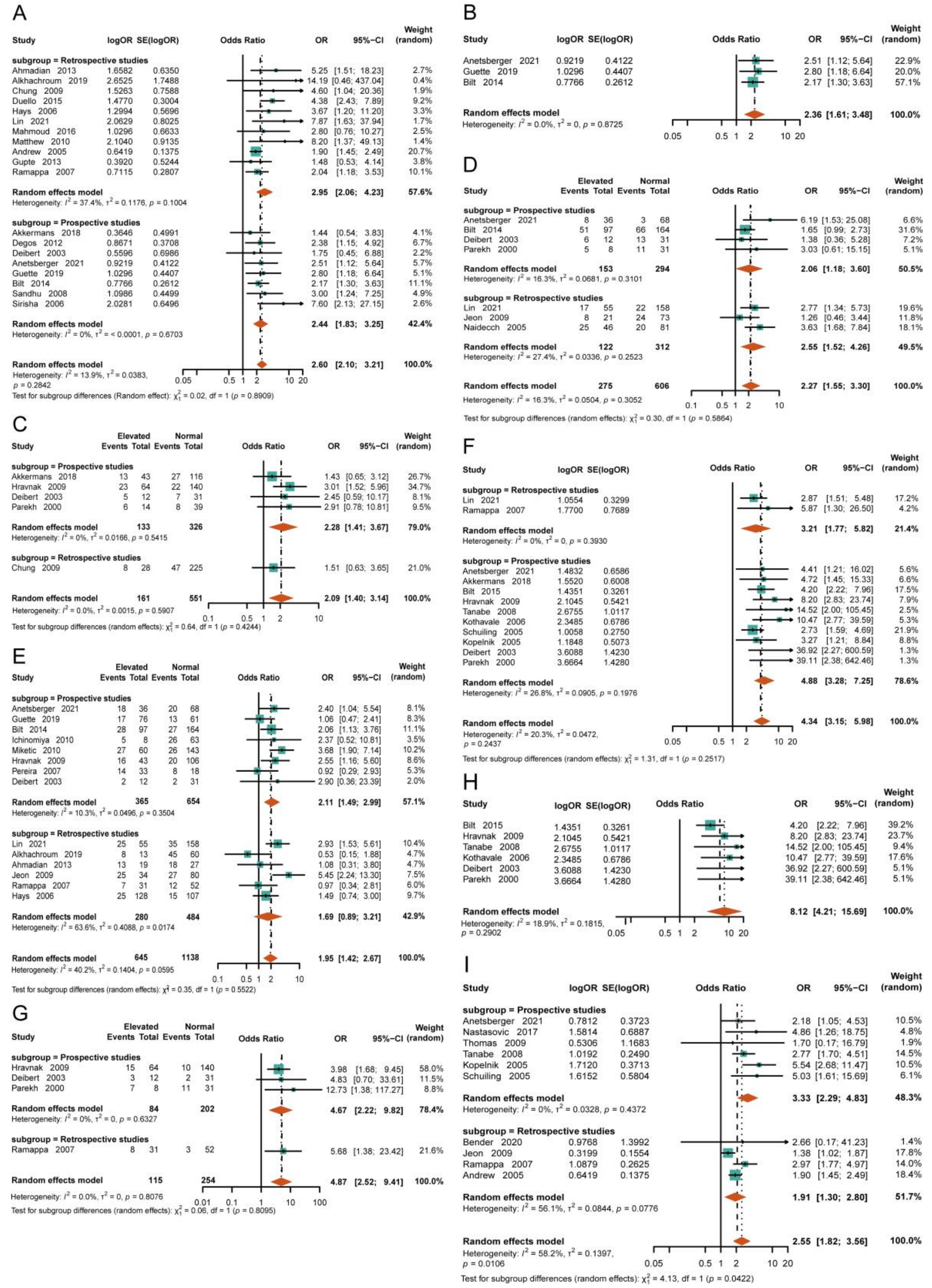

Figure S1. Forest plot of the relationship between troponin elevation and mortality, complications in patients with total subarachnoid hemorrhage

A. In-hospital mortality. B. Mortality three months after discharge. C. Disturbance of consciousness. D. Delayed cerebral ischemia or cerebral vasospasm. E. Disability. F. Cardiac dysfunction. G. Arrhythmia. H. Abnormal ventricular wall motion. I. Neurogenic pulmonary edema.

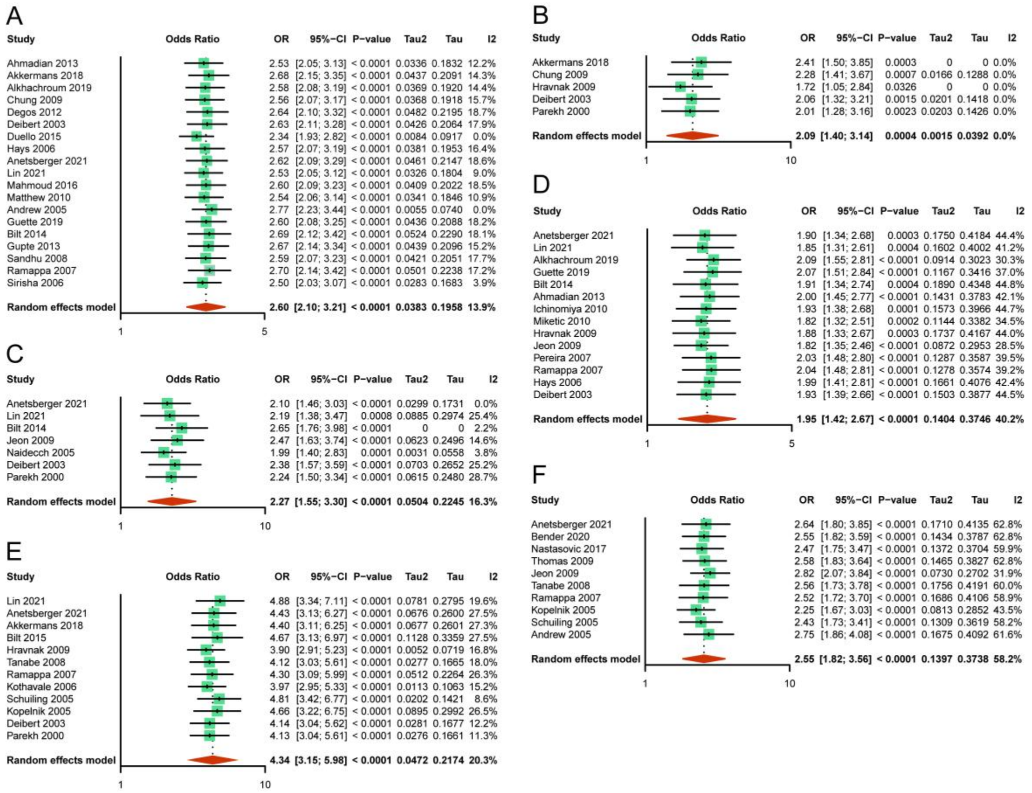

Figure S2. The Sensitivity analysis of the relationship between troponin elevation and mortality and complications in patients with subarachnoid hemorrhage (both aneurysmal and traumatic).

A. Mortality. B. Disturbance of consciousness. C. Delayed cerebral ischemia or cerebral vasospasm. D. Disability. E. Cardiac dysfunction. F. Neurogenic pulmonary edema.

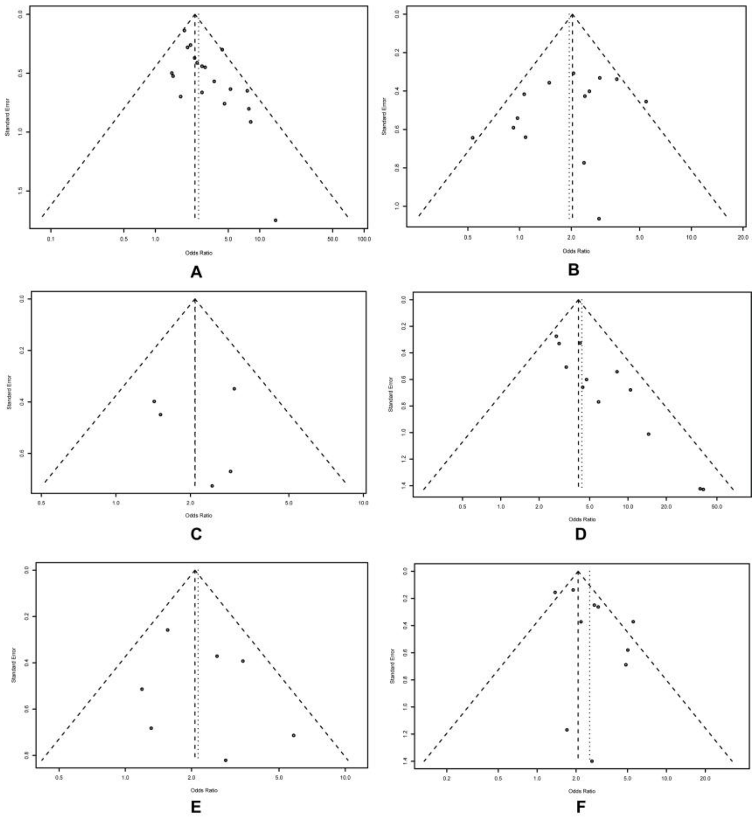

Figure S3. A. Funnel plot of mortality. B. Funnel plot of consciousness. C. Funnel plot of DCI. D. Funnel plot of disability rate. E. Funnel plot of cardiac dysfunction. F. Funnel of pulmonary edema.

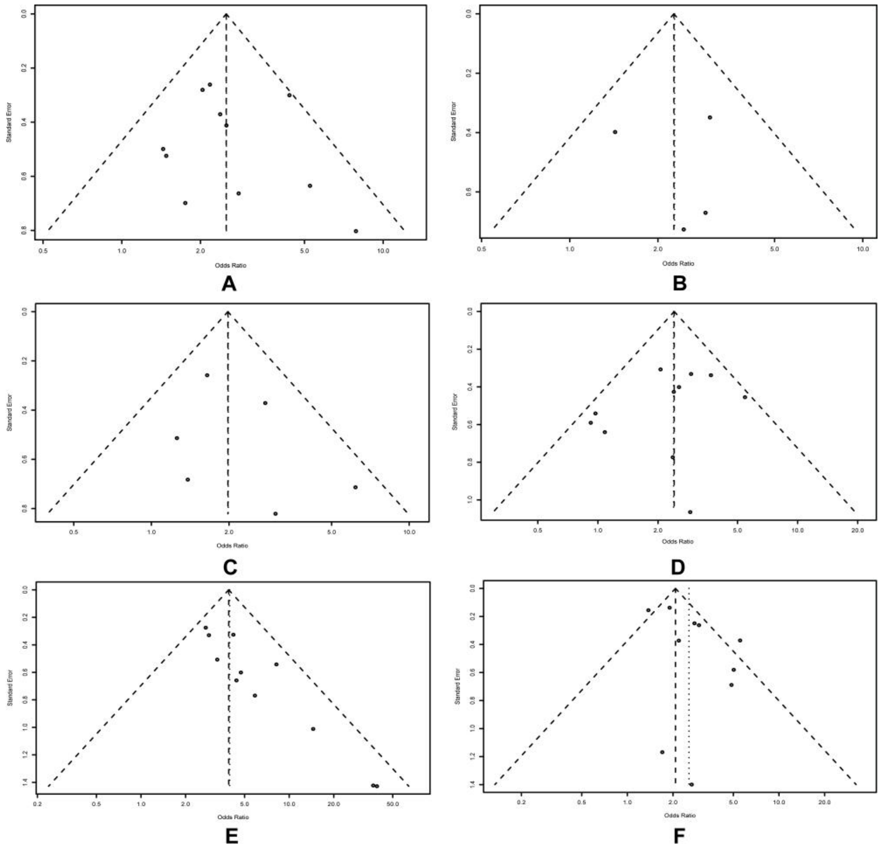

Figure S4. A. Funnel of mortality after aSAH. B. Funnel plot of levels of consciousness after aSAH. C. Funnel of delayed cerebral ischemia after aSAH. D. Funnel plot of disability after aSAH. E. Funnel of cardiac dysfunction after aSAH. F. Funnel of neurogenic pulmonary edema afer aSAH.
